# Does IQ predict total and cardiovascular disease mortality as strongly as other risk factors? Comparison of effect estimates using the Vietnam Experience Study

**DOI:** 10.1136/hrt.2008.149567

**Published:** 2008-12-10

**Authors:** G D Batty, M J Shipley, C R Gale, L H Mortensen, I J Deary

**Affiliations:** 1 MRC Social and Public Health Sciences Unit, University of Glasgow, Glasgow, UK; 2 MRC Centre for Cognitive Ageing and Cognitive Epidemiology, University of Edinburgh, Edinburgh, UK; 3 Department of Epidemiology and Public Health, University College London, London, UK; 4 MRC Epidemiology Resource Centre, University of Southampton, Southampton, UK; 5 National Institute of Public Health, University of Southern Denmark, Copenhagen, Denmark; 6 Department of Psychiatry and Behavioral Sciences, Duke University Medical Center, Durham, North Carolina, USA

## Abstract

**Objective:**

To compare the strength of the relation of two measurements of IQ and 11 established risk factors with total and cardiovascular disease (CVD) mortality.

**Methods:**

Cohort study of 4166 US male former army personnel with data on IQ test scores (in early adulthood and middle age), a range of established risk factors and 15-year mortality surveillance.

**Results:**

When CVD mortality (n = 61) was the outcome of interest, the relative index of inequality (RII: hazard ratio; 95% CI) for the most disadvantaged relative to the advantaged (in descending order of magnitude of the first six based on age-adjusted analyses) was: 6.58 (2.54 to 17.1) for family income; 5.55 (2.16 to 14.2) for total cholesterol; 5.12 (2.01 to 13.0) for body mass index; 4.70 (1.89 to 11.7) for IQ in middle age; 4.29 (1.70 to 10.8) for blood glucose and 4.08 (1.63 to 10.2) for high-density lipoprotein cholesterol (the RII for IQ in early adulthood was ranked tenth: 2.88; 1.19 to 6.97). In analyses featuring all deaths (n = 233), the RII for risk factors most strongly related to this outcome was 7.46 (4.54 to 12.3) for family income; 4.41 (2.77 to 7.03) for IQ in middle age; 4.02 (2.37 to 6.83) for smoking; 3.81 (2.35 to 6.17) for educational attainment; 3.40 (2.14 to 5.41) for pulse rate and 3.26 (2.06 to 5.15) for IQ in early adulthood. Multivariable adjustment led to marked attenuation of these relations, particularly those for IQ.

**Conclusions:**

Lower scores on measures of IQ at two time points were associated with CVD and, particularly, total mortality, at a level of magnitude greater than several other established risk factors.

There is growing interest in the association of mental ability test scores (referred to here as IQ) with chronic disease and other health outcomes.^1^ ^2^ In this field of cognitive epidemiology,^3^ investigators have examined the predictive ability of scores from mental tests administered to children and young adults. When these cohorts were followed up for as much as six decades, higher IQ was associated with a significantly reduced risk of all-cause mortality^2^ ^4^^–^6 and, in particular, cardiovascular disease (CVD).^4^ ^7^^–^10 Importantly, because IQ was ascertained in young people in these studies, these scores are likely to be premorbid.

This body of work represents a considerable advance on similar studies of middle- and older-aged people where the same relation has been reported.^11^ ^12^ Cross-sectional studies in these age groups have shown lower cognitive function in people with common chronic diseases such as diabetes^13^ and atherosclerosis^14^ relative to those without these conditions. Any apparently protective effects of higher cognitive ability against premature mortality and CVD might therefore be due to reverse causality, such that higher levels of comorbidity (clinical and subclinical) influence both cognition and mortality risk.

Within this emerging area of research, a judgment about the importance of the predictive validity of early life mental test scores versus other risk factors is needed. This can be achieved by comparing the magnitude of the association between mental ability and later mortality risk with that for well-established risk factors. Such risk factors include smoking, obesity and raised levels of blood pressure, cholesterol and blood glucose.^15^ However, because few studies hold such detailed data, no such comparison currently exists. The Vietnam Experience Study provides data which afford this comparison.

## METHODS

The Vietnam Experience Study, with particular emphasis on the use of IQ data, has been described in detail elsewhere.^6^ ^9^ ^16^ ^17^ In brief, the cohort was established in the early 1980s in order to examine the health of soldiers who had participated in the Vietnam war in comparison with those with other postings.^18^^–^21 In formulating this cohort, data were extracted retrospectively from the US army enlistment records of 18 313 former military personnel who had been drawn randomly from approximately five million such veterans who entered the service between 1965 and 1971. The average age of men at army entry was 20 years. The data available from these records were service income (based on military rank and using 1964 pay scales: US$83, US$86, US$99, US$120 and US$145), ethnicity (“white”, “black”, or “other”; the latter group comprising Hispanics, Asians, Pacific Islanders, American Indians and Alaskan Natives) and place of service (Vietnam vs “other”). Existing records also provided recruits’ IQ test result at army entry, as based on the Army General Technical Test (AGTT) within the Army Classification Battery.^22^ This battery consists of two subtests: verbal and arithmetic reasoning. Scores on the general technical section correlate highly with scores on standard tests of intelligence.^22^

In 1985–6, when recruits were around 38 years of age, 16 349 of the original 18 313 men were found to be both alive and of known whereabouts. In total, 15 288 (94%) of these participated in a telephone survey concerning their health and related behaviours. After this contact, a random sample of these respondents (n = 6443) was invited to attend a 3-day medical examination when more detailed data were collected; 4462 (69%) did so (see later for a discussion of the representativeness of this group). During this examination, blood cholesterol (including fractions), blood glucose, blood pressure, height and weight were measured using standard protocols. In short, after an overnight fast, blood was drawn and analysed for levels of triglycerides; and cholesterol fractions were ascertained using a Kodak Ektachem 700 autoanalyzer.^18^ ^19^ Serum glucose level was determined with a standard adaptation of the glucose oxidase–peroxidase–chromogen-coupled system for glucose determination in biological fluids.^18^ ^19^ With the participant in a sitting position, blood pressure was measured twice in the right arm using a standard mercury sphygmomanometer; for the purpose of analyses, an average was computed. Measured height (kg) and weight (m) were used to calculate body mass index based on the usual formula (weight/height^2^).

During the medical examination, alongside other psychological tests, the AGTT was re-administered by psychology technicians under the supervision of clinical psychologists. The Wechsler Adult Intelligence Scale,^23^ a comprehensive and widely used test of cognitive abilities, was also utilised. AGTT scores from both army entry and the medical examination in middle age were strongly correlated with information subtest scores and block design scores based on the Wechsler Adult Intelligence Scale (range for the Pearson correlation coefficients 0.51–0.74). With a total of four indicators of IQ, a priori, we elected to focus on the predictive value of the two administrations of the AGTT as these represent two distinct periods of the life course. Further, to rank the magnitude of all four measures of IQ against the classic risk factors could artificially inflate their importance. Throughout this manuscript we distinguish between the AGTT administered at army entry (“AGTT-1”) and in middle age (“AGTT-2”).

### Ascertainment of mortality

The vital status of men after the medical examination was ascertained by crosschecking against a variety of mortality databases supplied by the US army, the Veterans Administration (Beneficiary Identification and Records Locator Subsystem), the Social Security Administration, the Internal Revenue Service and the National Center for Health Statistics (National Death Index). CVD mortality was based on international classification of disease codes 390–434 and 436–45 (version 9) and I00–I99 (version 10).

### Statistical analyses

Given that different risk factors have different scales and some are continuous while others are discrete, a direct comparison of the magnitude of the relation of each with mortality is problematic. For this reason, we used the relative index of inequality (RII) to quantify risk factor–mortality associations, as we have done previously.^24^ The RII is derived by ranking the subjects on each of the risk factors and then dividing this score by the sample size to yield a value between 0 and 1. When included in Cox proportional hazard models with mortality as the outcome, the RII can be interpreted as the mortality hazard between the extreme ends of the risk factor distribution. Thus, an RII of 2.0 indicates that the mortality hazard between the extreme ends of the risk factor distribution is twice as high for the most disadvantaged (high risk) as for the most advantaged (low risk). In these Cox regression analyses, we adjusted for age, then other potentially confounding (eg, army income) and mediating (eg, occupational prestige, smoking) variables. Only the 4166 men who had complete data on all variables were included in the analyses. Follow-up time was taken from the medical examination until censoring, death or 31 December 2000, which ever came first.

## RESULTS

A total of 15.1 years of follow-up gave rise to 233 deaths (61 from CVD). Table 1 shows the RII for each of the nine risk factors in relation to total and CVD mortality. The association between risk factors and each mortality outcome is ordered in descending magnitude based on the age-adjusted analyses. Most of the variables examined were related to both total and CVD mortality in the expected directions; the only exceptions being total cholesterol and body mass index in the total mortality analyses where there was no evidence of an association.

**Table 1:** Relative index of inequality (hazard ratio*; 95% CI) for the relation of IQ and established risk factors with total and cardiovascular disease mortality (n = 4166)

Risk factors	Age	Age, army income, place of service and ethnicity	Multiple adjustment†
*Total mortality (233 deaths)*			
Family income	7.46 (4.54 to 12.3)	5.37 (3.21 to 8.98)	4.07 (2.33 to 7.11)
AGTT-2	4.41 (2.77 to 7.03)	2.66 (1.58 to 4.48)	1.03 (0.55 to 1.93)
Smoking	4.02 (2.37 to 6.83)	3.59 (2.11 to 6.11)	2.27 (1.31 to 3.95)
Educational attainment	3.81 (2.35 to 6.17)	3.05 (1.85 to 5.02)	1.81 (0.97 to 3.38)
Pulse rate	3.40 (2.14 to 5.41)	3.39 (2.14 to 5.37)	2.53 (1.57 to 4.08)
AGTT-1	3.26 (2.06 to 5.15)	1.80 (1.07 to 3.01)	0.67 (0.36 to 1.24)
Occupational prestige	3.02 (1.90 to 4.79)	2.24 (1.39 to 3.61)	1.05 (0.60 to 1.84)
Fasting blood glucose	1.69 (1.08 to 2.66)	1.58 (1.01 to 2.47)	1.41 (0.87 to 2.26)
Systolic blood pressure	1.66 (1.06 to 2.60)	1.45 (0.93 to 2.27)	1.37 (0.75 to 2.50)
HDL cholesterol	1.66 (1.06 to 2.60)	2.03 (1.29 to 3.20)	1.92 (1.18 to 3.10)
Diastolic blood pressure	1.59 (1.02 to 2.50)	1.34 (0.86 to 2.10)	1.03 (0.57 to 1.87)
Total cholesterol	1.07 (0.68 to 1.67)	1.07 (0.68 to 1.68)	0.91 (0.58 to 1.43)
Body mass index	0.91 (0.58 to 1.43)	0.91 (0.58 to 1.41)	0.61 (0.36 to 1.01)
			
*CVD mortality (61 deaths)*			
Family income	6.58 (2.54 to 17.1)	5.18 (1.92 to 14.0)	4.26 (1.45 to 12.5)
Total cholesterol	5.55 (2.16 to 14.2)	5.42 (2.11 to 13.9)	3.94 (1.52 to 10.2)
Body mass index	5.12 (2.01 to 13.0)	4.87 (1.93 to 12.3)	2.54 (0.87 to 7.43)
AGTT-2	4.70 (1.89 to 11.7)	3.21 (1.17 to 8.86)	1.23 (0.36 to 4.19)
Fasting blood glucose	4.29 (1.70 to 10.8)	3.97 (1.58 to 9.96)	2.19 (0.82 to 5.82)
HDL cholesterol	4.08 (1.63 to 10.2)	4.96 (1.97 to 12.5)	2.87 (1.06 to 7.76)
Occupational prestige	3.97 (1.61 to 9.82)	3.30 (1.30 to 8.39)	1.64 (0.54 to 4.91)
Smoking	3.96 (1.42 to 11.0)	3.85 (1.37 to 10.8)	2.82 (0.97 to 8.19)
Educational attainment	3.29 (1.30 to 8.32)	2.78 (1.07 to 7.21)	1.28 (0.38 to 4.32)
AGTT-1	2.88 (1.19 to 6.97)	1.75 (0.65 to 4.74)	0.53 (0.16 to 1.75)
Pulse rate	2.88 (1.17 to 7.09)	2.87 (1.17 to 7.04)	1.26 (0.49 to 3.22)
Systolic blood pressure	2.75 (1.13 to 6.69)	2.52 (1.04 to 6.12)	1.40 (0.43 to 4.59)
Diastolic blood pressure	2.31 (0.95 to 5.61)	2.07 (0.85 to 5.02)	0.78 (0.25 to 2.47)

*The hazard ratios show the mortality hazard of the most disadvantaged men with the most advantaged (that is, extreme ends of the risk factor distribution); †adjustment for all variables in the table with the exception of IQ-wave-1 and IQ-wave-2 which are not mutually adjusted.

AGTT-1, Army General Technical Test at army entry; AGTT-2, Army General Technical Test in middle age; CVD, cardiovascular disease; HDL, high-density lipoprotein.

When total mortality was the outcome of interest, in descending order of magnitude in age-adjusted analyses, family income in middle age (by some distance, the strongest predictor), AGTT-2, smoking, educational attainment, pulse rate, AGTT-1 and occupational prestige were the risk factors most strongly related to mortality risk, whereby the most disadvantaged men experienced over three times the risk of death relative to the advantaged. These effect estimates were followed in magnitude by a group of risk factors which were associated with somewhat less than double the mortality hazard. Attenuation of these associations was apparent when we added army income, place of service and ethnicity to the multivariable model. However, in general, similar rankings were apparent: again, low income was associated with over five times the risk of mortality in comparison with the higher end of this continuum, while several risk factors then clustered around a tripling of mortality risk. Multiple control for all variables, which included markers of socioeconomic position, led to further attenuation, with the association of both IQ variables with mortality lost, as it was for other selected risk factors. In age-adjusted analyses (RII; 95% CI), both the information subtest scores (3.51; 2.20 to 5.61) and block design (5.40; 3.35 to 8.72) scores based on the Wechsler Adult Intelligence Scale were strongly related to total mortality whereby men with lower scores experienced higher risk (detailed results available upon request).

Most of these risk ratios showed stronger effect estimates when CVD was the outcome of interest. In descending order of risk in age-adjusted analyses, family income, total cholesterol, body mass index, AGTT-2, fasting blood glucose, high-density lipoprotein (HDL) cholesterol, occupational prestige and smoking were most strongly related to CVD mortality rates, such that the most disadvantaged men experienced around four or more times the risk of death relative to the advantaged. Again, adjustment for confounding factors led to attenuation of the association with CVD mortality for the risk indices. Additional attenuation was apparent after multiple control and, again, the relations for both IQ variables with CVD mortality was lost. Once more, both the information subtest (4.91; 1.95 to 12.4) and block design (4.90; 1.95 to 12.3) scores based on the Wechsler Adult Intelligence Scale were strongly related to CVD mortality (detailed results not shown but available upon request).

## DISCUSSION

The objective of this study was, for the first time, to compare the relative strength of the relation between IQ and mortality with the associations apparent for better established risk factors. The main finding was that, in age-adjusted analyses, lower IQ scores in both early adulthood and middle age were related to total and CVD mortality at a level of magnitude greater than many traditional risk indices. Attenuation of the association of IQ with the mortality outcomes was most evident after mutual adjustment for all explanatory variables. As we have previously outlined,^6^ it may be that education, occupational prestige and income—which were included as explanatory factors in these analyses—are part of a pathway linking IQ with mortality, whereby high IQ scores result in educational achievement, placement into well-paid employment, enhanced social status and the accompanying benefits to health associated with the last of these. Alternatively, it is also plausible that educational attainment may represent at least a partial proxy for individual differences in IQ, as may also be the case for military rank. As such, including education in our statistical models may be seen as overadjustment—in the present cohort (*r* = 0.55), education and mental ability are closely related. We may therefore have underestimated the true predictive capacity of IQ in the multiply adjusted models.

We found that IQ in middle age was more strongly related to mortality risk than IQ when measured by the same test (AGTT) in early adulthood. IQ may be at least partially a “record” of bodily insults or pathology across the life course.^25^ Thus, as described, IQ scores have been shown to be inversely correlated with important chronic diseases, such as diabetes^13^ and atherosclerosis,^14^ conditions which may be subclinical but nonetheless increase mortality risk. By the time of the later IQ assessment, the prevalence of such comorbidities will have been higher than earlier in life. It may be, therefore, that low IQ test scores at this age already reflect deteriorating health.

There are, however, other equally plausible explanations for the apparently protective effect of higher IQ scores against premature mortality and CVD. We have previously outlined five^2^: (*a*) adult socioeconomic advantage (higher IQ leads to educational success, high social status and well-remunerated employment); (*b*) improved disease and injury prevention (higher IQ leads to behaviours more conducive to health, such as not smoking, higher levels of physical activity, prudent diet, seat belt use and so on); (*c*) better disease/injury management (high IQ results in the better management of serious illness/injury such as heart disease or head trauma via increased uptake of appropriate healthcare); (*d*) reduced psychiatric disease (people with a high IQ score have a lower risk of later psychiatric illness and therefore subsequent mortality) and (*e*) “body system integrity” (just as high IQ might reflect superior neural connections, it may also denote other optimally functioning physiological systems). It was beyond the realms of the present manuscript to explore these explanations; this will be the aim of future analyses of the present dataset.

This study has a number of strengths, including the wide range of risk factor data collected and the complete mortality follow-up. We chose to compare the risk factors using RIIs, which provide readily interpretable mortality estimates that compare the extremes of the risk factor distribution. Similar results to those described would be obtained had we chosen to compare the risk factors using z scores. These would have expressed the mortality outcomes associated with a one standard deviation change in the risk factor, which is not easily interpreted when the risk factor is discrete with only two or three categories.

This study is not, however, without its shortcomings. First, the present analyses are based on a sample of men with complete information on IQ, covariates at telephone interview and medical examination and mortality. This group represents 67% of the subsample invited to the medical examination—that is, 26% of people originally enrolled in the study.^6^ Although the latter is based on a random sample of surviving men, concerns are nonetheless raised about selection bias—that is, if the reported results differ markedly between people included in the analyses and those not. We have previously tested this directly by relating IQ scores ascertained at wave one with total mortality in men included in this analytical sample and, separately, those who were not.^6^ The strength of this association (hazards ratio per one SD increase in IQ; 95% CI) was similar in men included (0.71; 0.63 to 0.81) and those excluded (0.79; 0.75 to 0.84) from the analyses (p value for difference  =  0.22).^6^ A second limitation is that because the risk factor data were mainly collected in the mid-1980s we do not have information on contemporary and emerging predictors such as inflammatory and thrombotic markers. Third, genetic factors in their own right and also via their interaction with environmental exposures, are increasingly being implicated in the aetiology of intelligence.^26^ This study did not, unfortunately, capture any genetic material.

In conclusion, in this, the first study to examine the relative strengths of IQ and established risk factors as predictors of total and CVD mortality, there was some evidence that low IQ was among the more powerful. Further examination is required.
